# Supplementary material for: PGC-1α mediates a metabolic host defense response in human airway epithelium during rhinovirus infections
Source: Nat Commun. 2021 Jun 16;12:3669. doi: 10.1038/s41467-021-23925-z (PMC8209127; doi:10.1038/s41467-021-23925-z)
Supplement: Supplementary file 1 — Supplemental Information [file 41467_2021_23925_MOESM1_ESM.pdf]

Supplementary Information

PGC-1α mediates a metabolic host defense response in human airway epithelium during rhinovirus infections

Aubrey N. Michi[1,2], Bryan G. Yipp[2,3], Antoine Dufour[1,4], Fernando Lopes[5,6], David Proud[1,2]*

[1]Department of Physiology and Pharmacology, Cumming School of Medicine, University of Calgary, Calgary, AB, T2N 4N1 Canada

[2]Snyder Institute for Chronic Diseases, University of Calgary, Calgary, AB, T2N 4N1 Canada

[3]Department of Critical Care Medicine, Cumming School of Medicine, University of Calgary, Calgary, AB, T2N 4N1 Canada

[4]McCaig Institute for Bone and Joint Health, University of Calgary, Calgary, AB, T2N 4N1 Canada

[5]Institute of Parasitology, McGill University, Ste-Anne-de-Bellevue, QC, H9X 3V9, Canada

[6]Department of Microbiology and Immunology, McGill University, Montreal, QC, H3A 2B4, Canada

*Correspondence: dproud@ucalgary.ca

Supplementary Figure 1

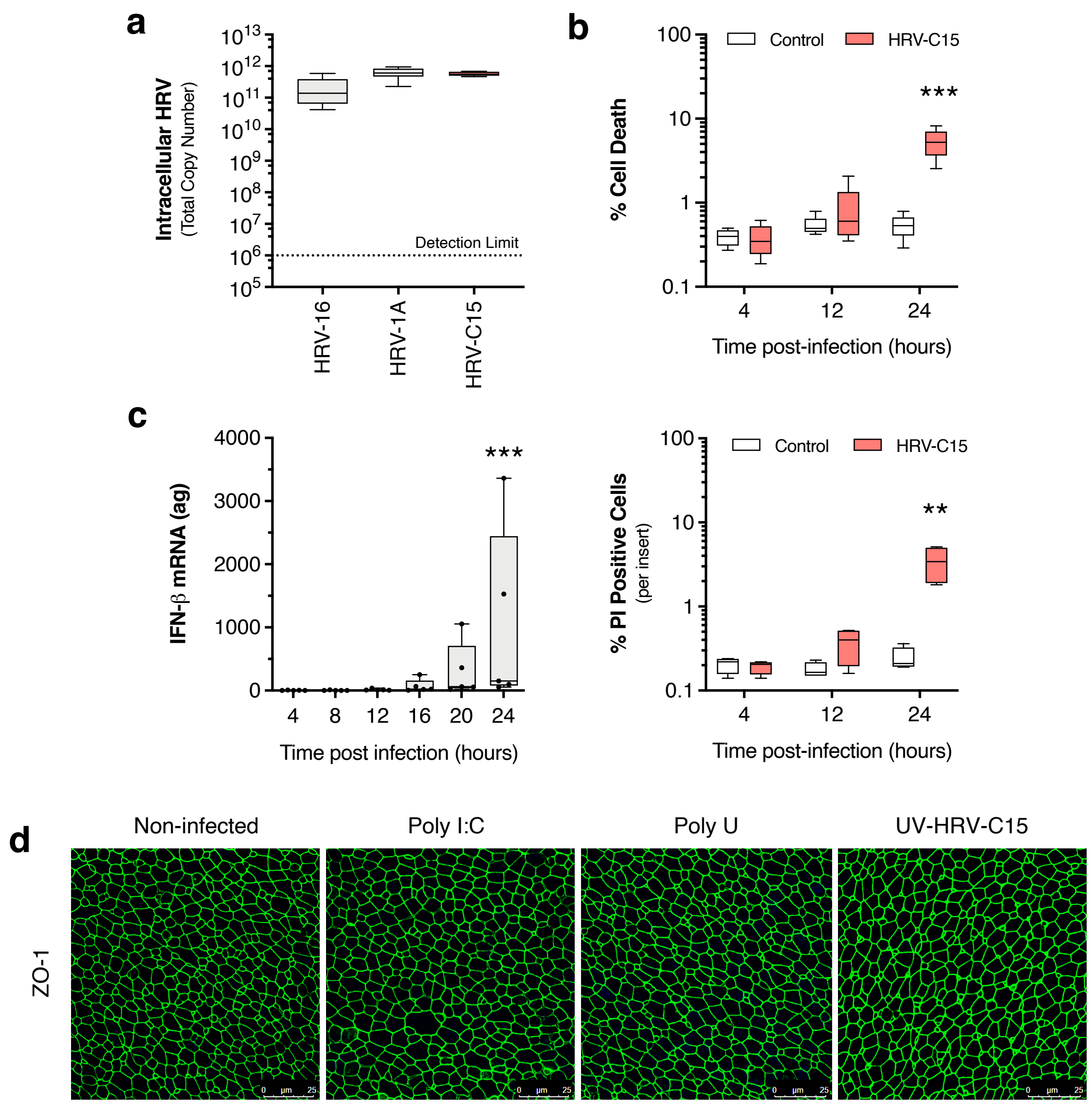

Supplementary Figure 1. Effects of Live HRV-C15 Infection in ALI Cultures

(a) Matched intracellular HRV RNA copy number from HRV strain comparison in Figure 1b, quantified by RT-PCR. Dashed line represents RT-PCR detection limit. There was no detectable HRV in non-infected ALI cultures. Data represented as boxplots and analyzed by one-way ANOVA with Holm-Sidak multiple comparisons, n=6 donors.

(b) Apical and basolateral release of lactate dehydrogenase (LDH) to assess necrosis compared to a fully lysed matched donor (top). *In situ* live cell propidium iodide (PI) staining in ALI cultures at each infection time point (bottom). Quantified by 10 fields of view per condition per time point per donor. Data represented as boxplots and analyzed by two-way ANOVA with Holm-Sidak multiple comparisons: LDH ***p=0.0001, PI **p=0.0014, n=5 donors.

(c) Matched IFN-β mRNA from Figure 1h, quantified by RT-PCR. Data represented as boxplots and analyzed by one-way ANOVA with Dunnett multiple comparisons: **p=0.003, n=5 donors.

(d) Representative immunofluorescence image of ZO-1 (green) organization from Figure 1c. Scale bar, 10 μm.

Data represented as boxplots indicate the median (center line), upper and lower box bounds (IQR = first and third quartiles), and whiskers (min and max values), with individual donor data points superimposed onto the boxplot.

Supplementary Figure 2

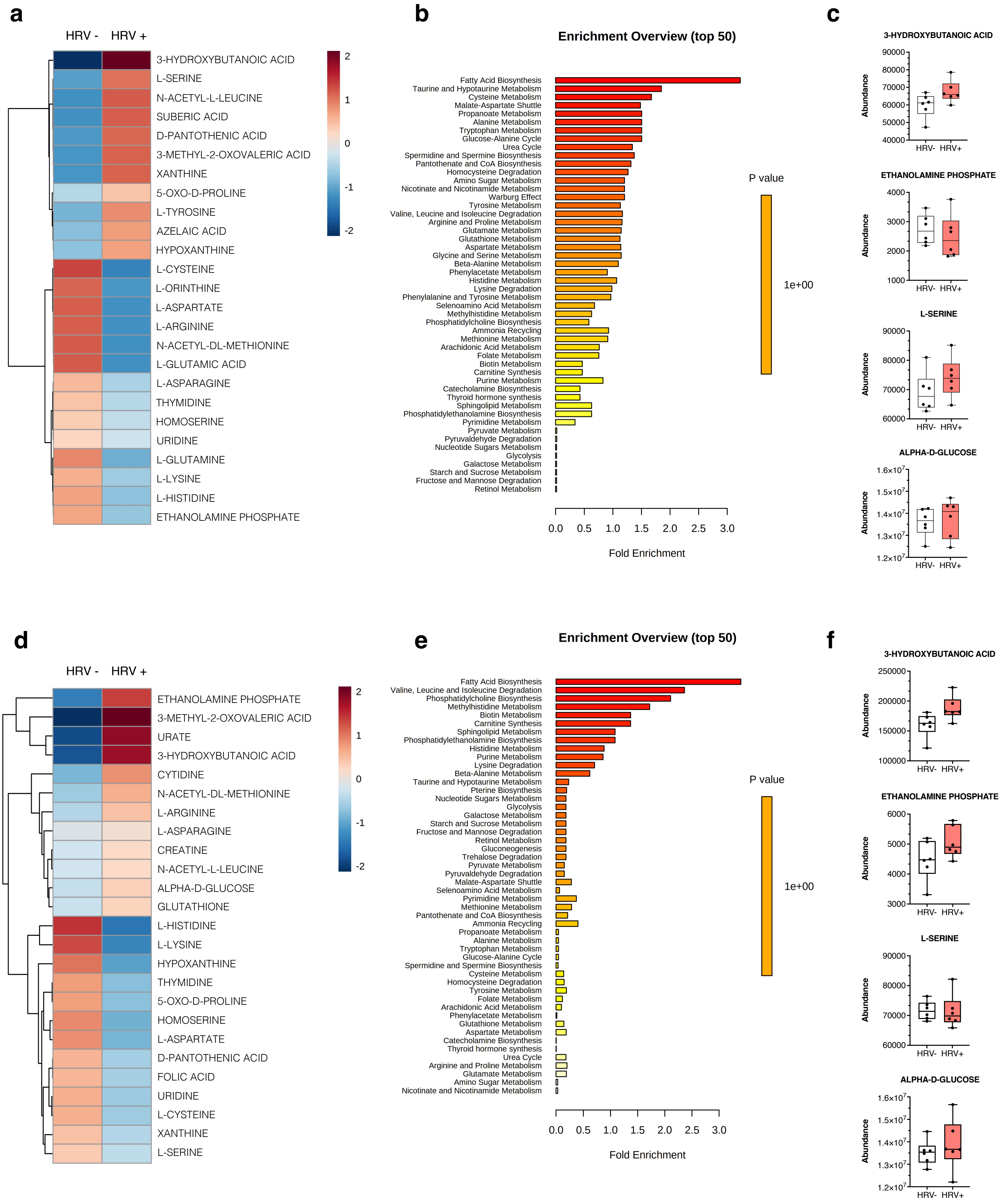

Supplementary Figure 2. Early HRV-C15 Infection Metabolomic Analysis

(a) Hierarchical Clustering Heatmap depicting group averages of metabolite concentration (Top 25) of HRV-C15 (HRV+) and non-infected (HRV-) ALI cultures at 4 h post-infection normalized to HRV- group. Distance measure: Euclidean. Clustering Algorithm: Ward. n=6 donors.

(b) Enrichment analysis of upregulated metabolites computed by Hits/Expected, (hits = observed hits; expected = expected hits) of HRV-C15 (HRV+) and non-infected (HRV-) ALI cultures at 4 h post-infection normalized to HRV- group. Data analyzed by MetaboAnalyst 4.0, Small Molecule Pathway Database (SMPDB) metabolite set library, n=6 donors.

(c) 3-Hydroxybutanoic acid, ethanolamine phosphate, L-serine and alpha-D-glucose abundance at 4 h post-infection measured by LC-MS. Data represented as boxplots and analyzed by two-tailed paired *t* test. Data not significant n=6 donors.

(d) Hierarchical Clustering Heatmap depicting group averages of metabolite concentration (Top 25) of HRV-C15 (HRV+) and non-infected (HRV-) ALI cultures at 12 h post-infection normalized to HRV- group. Distance measure: Euclidean. Clustering Algorithm: Ward. n=6 donors.

(e) Enrichment analysis of upregulated metabolites computed by Hits/Expected, (hits = observed hits; expected = expected hits) of HRV-C15 (HRV+) and non-infected (HRV-) ALI cultures at 12 h post-infection normalized to HRV- group. Data analyzed by MetaboAnalyst 4.0, Small Molecule Pathway Database (SMPDB) metabolite set library, n=6 donors.

(f) 3-Hydroxybutanoic acid, ethanolamine phosphate, L-serine and alpha-D-glucose abundance at 12 h post-infection determined by LC-MS. Data represented as boxplots and analyzed by two-tailed paired *t* test. Data not significant, n=6 donors.

Data represented as boxplots indicate the median (center line), upper and lower box bounds (IQR = first and third quartiles), and whiskers (min and max values), with individual donor data points superimposed onto the boxplot.

Supplementary Figure 3

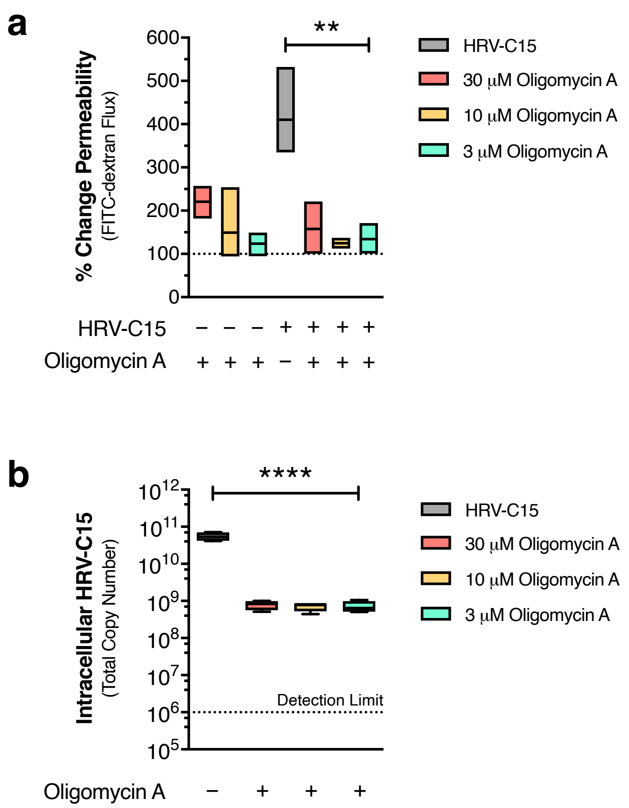

Supplementary Figure 3. Effects of Oligomycin A on Barrier Function and HRV-C15 Titers

(a) Oligomycin A dose response measuring FITC-dextran permeability with and without HRV-C15 infection. Data represented as minimum to maximum floating bars indicating mean (center line) of percent change from non-infected control (dashed line). Data analyzed by one-way ANOVA with Holm-Sidak for multiple comparisons: **p=0.002 for all comparisons to HRV-C15 alone, n=3 donors.

(b) Matched intracellular HRV-C15 titer from Supplementary Figure 3a. Dashed line represents RT-PCR detection limit. There was no detectable HRV-C15 in non-infected cultures. Data represented as boxplots and analyzed by one-way ANOVA with Holm-Sidak for multiple comparisons: ****p<0.0001 for all comparisons to HRV-C15 alone, n=4 donors.

Data represented as boxplots indicate the median (center line), upper and lower box bounds (IQR = first and third quartiles), and whiskers (min and max values).

Supplementary Figure 4

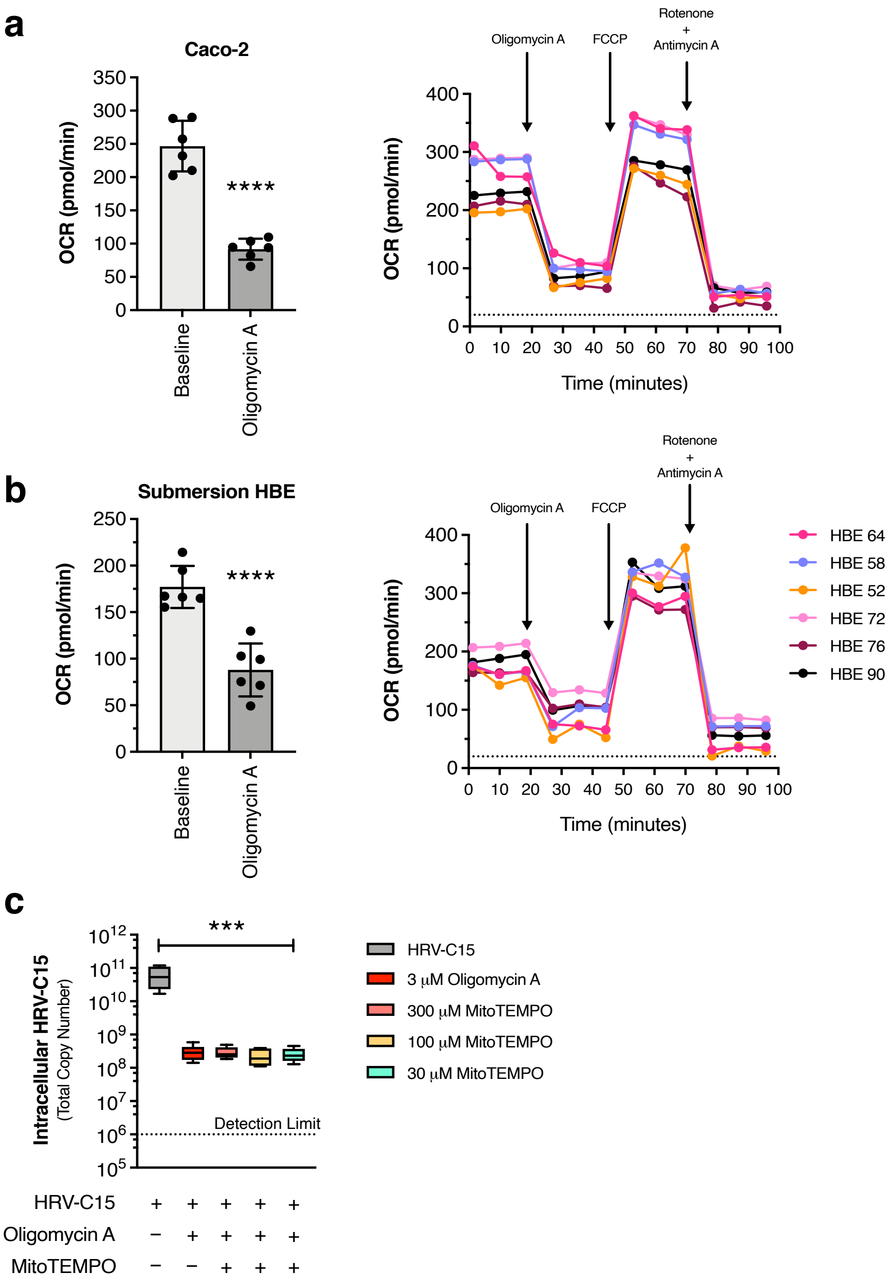

Supplementary Figure 4. Oligomycin A Effect on Oxygen Consumption Rate in Other Epithelial Cell Models

(a) Caco-2 monolayer metabolic performance assessed by mitochondrial stress test in a Seahorse XFe24 Analyzer. Data represented (left) as mean ± SD and matched biological replicate performance (right) and analyzed by two-tailed paired *t* test: ****p<0.0001, n=6.

(b) Non-differentiated primary human bronchial epithelial (HBE) monolayers (ALI progenitor cells) metabolic performance assessed by mitochondrial stress test in a Seahorse XFe24 Analyzer. Data represented (left) as mean ± SD and matched individual donor performance (right) and analyzed by two-tailed paired *t* test: ****p<0.0001, n=6 donors.

(c) Matched intracellular HRV-C15 RNA copy number from Figure 5d of MitoTEMPO dose response in combination with 3 μM oligomycin A. Dashed line represents RT-PCR detection limit. There was no detectable HRV-C15 in non-infected ALI cultures. Data represented as boxplots and analyzed by one-way ANOVA with Holm-Sidak for multiple comparisons: ***p=0.0003 for all comparisons to HRV-C15 alone, n=6 donors.

Data represented as boxplots indicate the median (center line), upper and lower box bounds (IQR = first and third quartiles), and whiskers (min and max values), with individual donor data points superimposed onto the boxplot.

Supplementary Figure 5

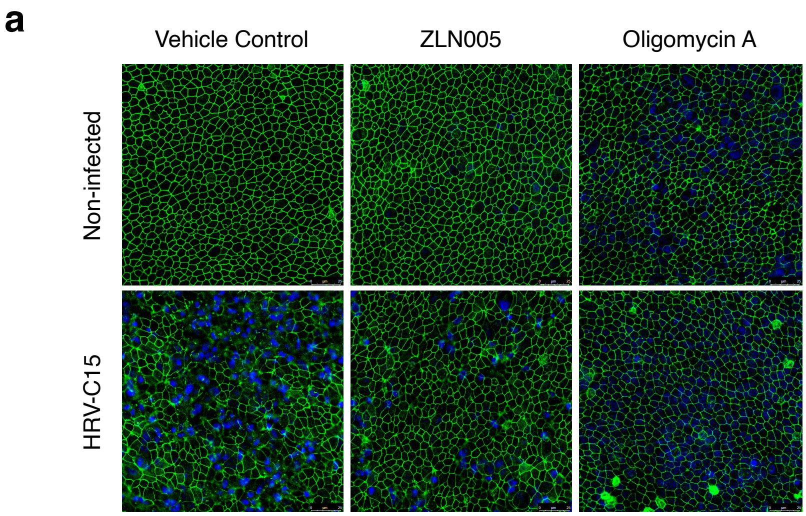

Supplementary Figure 5. Tight Junction Organization Post-Metabolic Intervention at 24 hours

(a) Representative immunofluorescence images of occludin (green) and nuclei (blue) matched from experiment in Figure 7d at 24 h post-infection. Representative confocal images were obtained from the same three independent experiments in Figure 7d performed using ALI cultures derived from three different lung donors and selected from 10 fields per condition per donor, n=3 donors. Scale bar, 25 μm.

Supplementary Table 1

Donor ID	Age	Sex	Ethnicity	Cause of Death
**32**	20	Male	Caucasian	Head Trauma
**44**	45	Female	Caucasian	Intracerebral Hemorrhage
**46**	54	Male	Caucasian	Head Trauma/Anoxia
**52**	20	Male	Caucasian	Head Trauma
**53**	63	Male	Caucasian	Intracerebral Hemorrhage
**55**	62	Female	Caucasian	Intracerebral Hemorrhage
**58**	13	Male	Caucasian	Head Trauma
**64**	38	Female	Caucasian	Head Trauma
**72**	62	Male	Caucasian	Intracerebral Hemorrhage
**74**	22	Male	African American/Black	Head Trauma
**76**	17	Female	Caucasian	Asphyxiation
**78**	60	Male	Hispanic/Latino	Head Trauma
**82**	42	Male	Caucasian	Intracerebral Hemorrhage
**84**	59	Male	Caucasian	Head Trauma
**85**	62	Female	Caucasian	Intracerebral Hemorrhage
**86**	33	Male	Caucasian	Head Trauma
**90**	43	Male	African American/Black	Intracerebral Hemorrhage
**91**	57	Female	Caucasian	Intracerebral Hemorrhage
**93**	65	Male	Caucasian	Intracerebral Hemorrhage
**97**	32	Male	Caucasian	Head Trauma
**98**	59	Female	Caucasian	Intracerebral Hemorrhage

Supplementary Table 1. Human Lung Donor Demographics

Bronchial epithelial cells were obtained from non-transplanted healthy human lungs and were used to generate all ALI cultures. Donors were non-smokers, had no known lung diseases, died of non-pulmonary causes, and had no pulmonary trauma. Donors of any age, sex and race were accepted provided they fulfilled these criteria. Each experimental replicate was performed using ALI cultures derived from one donor such that 1 donor = 1 biological replicate (n=1).

Supplementary Table 2

Supplementary Table 2. Primer and Probe Sequences

Human Gene	Forward Sequence 5' → 3'	Reverse Sequence 5' → 3'	TaqMan Probe Sequence (FAM/MGB) 5' → 3'
IFNL1	CACAGGAGCTAGCGAGCTTCA	TTTTCAGCTTGAGTGACTCTTCCA	AAGGCCAGGGACGC
IFNB1	GCAATTGAATGGGAGGCTTG	TCCTCAGGGATGTCAAAGTTCAT	TACTGCCTCAAGGACA
RSAD2	CCTGCTTGGTGCCTGAATCT	GCGCATATATTCATCCAGAATAAGG	ACCAGAAGATGAAAGACT
RHINOVIRUS	F_1: AGCCTGCGTGGCTGCC<br>F_2: AGCCTGCGTGGCGGC	R_1: ACACCCAAAGTAGTCGGTCCC<br>R_2: ACACCCAAAGTAGTTGGTCCCA	TCCGGCCCCTGAAT
GAPDH	Proprietary: Hs99999905		20X Gene Expression Kit
SLC2A1	Proprietary: Hs00892681		20X Gene Expression Kit
HK1	Proprietary: Hs00175976		20X Gene Expression Kit
HK2	Proprietary: Hs00606086		20X Gene Expression Kit
TFAM	Proprietary: Hs00273372		20X Gene Expression Kit
POLG	Proprietary: Hs00160298		20X Gene Expression Kit
PPARGC1A	Proprietary: Hs00173304		20X Gene Expression Kit

Supplementary Table 3

Supplementary Table 3. Resources and Reagents

RESOURCE	SUPPLIER	IDENTIFIER
**ANTIBODIES**		
mouse anti-occludin	Invitrogen (Carlsbad, CA, USA)	33-1500
mouse anti-ZO-1	Invitrogen (Carlsbad, CA, USA)	33-9100
rabbit anti-ZO-1	Invitrogen (Carlsbad, CA, USA)	40-2200
mouse anti-dsRNA	Scicons (Budapest, Hungary)	J2-1504
mouse anti-β-tubulin	MilliporeSigma (Burlington, MA, USA)	T0198
rabbit anti-β-tubulin	Cell Signaling Technology (Danvers, MA, USA)	9F3
rabbit anti-PGC-1α	Cell Signaling Technology (Danvers, MA, USA)	2178
mouse anti-GAPDH	AbD Serotec	MCA 4739
goat anti-mouse IgG (H+L) (AlexaFluor488)	Invitrogen (Carlsbad, CA, USA)	A11029
goat anti-rabbit IgG (H+L) (AlexaFluor488)	Invitrogen (Carlsbad, CA, USA)	A11034
goat anti-mouse IgG (H+L) (AlexaFluor647)	Invitrogen (Carlsbad, CA, USA)	A32728
HRP-linked anti-rabbit IgG	Cell Signaling Technology (Danvers, MA, USA)	7404
HRP-linked goat anti-mouse IgG	Jackson ImmunoResearch Laboratories (West Grove, USA)	115-035-003
Biotinylated horse anti-mouse IgG	Vector Laboratories (Burlingame, CA, USA)	PK-4002
**CELL CULTURE REAGENTS**		
Pronase	MilliporeSigma (Burlington, MA, USA)	53702
T75 cm^2 tissue culture flask	Corning (Corning, NY, USA)	430641U
PneumaCult-EX Basal Medium	StemCell Technologies (Vancouver, BC, Canada)	05009
50X Supplement	StemCell Technologies (Vancouver, BC, Canada)	05019
hydrocortisone	StemCell Technologies (Vancouver, BC, Canada)	07904
Fluconazole	MilliporeSigma (Burlington, MA, USA)	F8929
Penicillin/Streptomycin	Life Technologies (Carlsbad, CA, USA)	15140-122
TrypLE Select	Life Technologies (Carlsbad, CA, USA)	12563-011
12-well, 0.4 µm Transwells	Corning (Corning, NY, USA)	3460
Bovine Collagen Type I/III	Advanced BioMatrix (San Diego, CA, USA)	5005-B
PneumaCult ALI-Basal Medium	StemCell Technologies (Vancouver, BC, Canada)	05002
10X Supplement	StemCell Technologies (Vancouver, BC, Canada)	05003
100X Maintenance Supplement	StemCell Technologies (Vancouver, BC, Canada)	05006
0.2 % heparin solution	StemCell Technologies (Vancouver, BC, Canada)	07980
Dulbecco's PBS (DPBS)	Invitrogen (Carlsbad, CA, USA)	14190-144
HEPES (1 M)	Invitrogen (Carlsbad, CA, USA)	15630-080
F12	Invitrogen (Carlsbad, CA, USA)	11765-05
DMEM	Invitrogen (Carlsbad, CA, USA)	11885-084
Fetal Bovine Serum (FBS)	Invitrogen (Carlsbad, CA, USA)	16000-044
Seahorse XFe24 Flux Pak	Agilent Technologies (Santa Clara, CA, USA)	102340-100
Seahorse XF DMEM Medium	Agilent Technologies (Santa Clara, CA, USA)	103575-100
Seahorse XF 1 M Glucose Solution	Agilent Technologies (Santa Clara, CA, USA)	103577-100
Seahorse XF 100 mM Pyruvate Solution	Agilent Technologies (Santa Clara, CA, USA)	103578-100
Seahorse XF 200 mM Glutamine Solution	Agilent Technologies (Santa Clara, CA, USA)	103579-100
HBSS	Invitrogen (Carlsbad, CA, USA)	14175-095
Gentamicin	Invitrogen (Carlsbad, CA, USA)	15750-060
Leibovitz's L15	Invitrogen (Carlsbad, CA, USA)	11415-064
Dimethyl Sulfoxide (DMSO)	MilliporeSigma (Burlington, MA, USA)	D2650
MEM non-essential amino acids	Invitrogen (Carlsbad, CA, USA)	11140-050
L-glutamine	Invitrogen (Carlsbad, CA, USA)	25030-081
**CELL LINES**		
H1 HeLa	American Type Culture Collection (Manassas, VA, USA)	CRL-1958
WI-38	American Type Culture Collection (Manassas, VA, USA)	CCL-75
Caco-2	American Type Culture Collection (Manassas, VA, USA)	HTB-37
**RHINOVIRUSES**		
HRV-16 (strain 11757)	American Type Culture Collection (Manassas, VA, USA)	VR-283
HRV-1A (strain 2060)	American Type Culture Collection (Manassas, VA, USA)	VR-1559
HRV-C15 (pC15-RZ)	Dr. James Gern (University of Wisconsin, Madison, WI, USA)	-

Supplementary Table 3 (continued)

Supplementary Table 3. Resources and Reagents

RESOURCE	SUPPLIER	IDENTIFIER
**COMMERCIAL ASSAYS**		
CytoTox96 Non-Radioactive Cytotoxicity Assay	Promega (Madison, WI, USA)	G1780
BioRad DC Protein Assay	BioRad Laboratories (Mississauga, ON, Canada)	5000112
QIAmp Viral RNA Mini Kit	Qiagen (Mississauga, ON, Canada)	52904
NucleoSpin RNA Kit	Macherey-Nagel, GmbH & Co (Duren, Germany)	740955
Mass Spectrometry Metabolite Library	IROA Technologies (Bolton, MA, USA)	MSMLS
**REAGENTS AND CHEMICALS**		
ZLN005	Cayman Chemical (Ann Arbor, MI, USA)	14121
SR18292	Cayman Chemical (Ann Arbor, MI, USA)	22084
Oligomycin A	Tocris (Bristol, UK)	4110
2-Deoxy-D-glucose (2-DG)	MilliporeSigma (Burlington, MA, USA)	D8375
MitoTEMPO	Cayman Chemical	16621
Paraformaldehyde, 16% w/v	Alfa Aesar (Haverhill, MA, USA)	43368
Bovine Serum Albumin (BSA)	MilliporeSigma (Burlington, MA, USA)	A7906
TritonX-100	MilliporeSigma (Burlington, MA, USA)	T9284
Superfrost Plus, glass slides	VWR (Radnor, PA, USA)	48311-703
coverglass (24 x 50)	ThermoFisher Scientific (Hampton, NH, USA)	12-545F
FluorSave Mounting Reagent	MilliporeSigma (Burlington, MA, USA)	345789
4',6-diamidine-2'-phenylindole dihydrochloride (DAPI)	Invitrogen (Carlsbad, CA, USA)	D1306
Methanol (MeOH)	MilliporeSigma (Burlington, MA, USA)	34860
BstBI restriction enzyme	New England BioLabs (Ipswich, MA, USA)	R0519L
T7 RiboMAX Large Scale RNA System	Promega (Madison, WI, USA)	P1300
Lipofectamine RNAiMax	Invitrogen (Carlsbad, CA, USA)	13778-150
CellROX Green Reagent (488nm)	Invitrogen (Carlsbad, CA, USA)	C10444
Hoechst 33342	Invitrogen (Carlsbad, CA, USA)	H1399
Propidium Iodide	Invitrogen (Carlsbad, CA, USA)	P1304MP
Avidin/Biotin Blocking Kit	Vector Laboratories (Burlingame, CA, USA)	SP-2001
VECTASTAIN ABC Peroxidase Kit	Vector Laboratories (Burlingame, CA, USA)	PK4002
3,3'-diaminobenzidine (DAB)	Vector Laboratories (Burlingame, CA, USA)	SK4100
Gills II Hematoxylin	Leica Biosystems (Wetzlar, Germany)	3801522
Permount	Fischer Scientific (Waltham, MA, USA)	SP15-500
Pierce ECL Substrate	Thermo Scientific (Waltham, MA, USA)	32106
FastStart Universal Probe Master (ROX)	Roche (Basel, Switzerland)	4914058001
Fluorescein Isothiocyanate (FITC)-dextran	Sigma (St. Louis, MI, USA)	FD4
TMTsixplex Isobaric Labeling Reagents	Thermo Scientific (Waltham, MA, USA)	90061
5X All-In-One RT MasterMix	Applied Biological Materials (Richmond, BC, Canada)	G490
10% Neutral Buffered Formalin (NBF)	MilliporeSigma (Burlington, MA, USA)	R03379-82
Surgipath cassettes	Leica Biosystems (Wetzlar, Germany)	3802631
Paraplast (paraffin wax)	Leica Biosystems (Wetzlar, Germany)	39601006
SeaKem, GTG Agarose	Lonza (Basel, Switzerland)	50070
Hydrogen peroxide (30% w/w)	MilliporeSigma (Burlington, MA, USA)	H1009
**EQUIPMENT**		
Q Exactive™ HF Hybrid Quadrupole-Orbitrap™ Mass Spec	Thermo Scientific (Waltham, MA, USA)	IQLAAEGAAPFALGMBFZ
Vanquish™ UHPLC System	Thermo Scientific (Waltham, MA, USA)	IQLAAAGABHFAPUMZZZ
Syncronis™ HILIC UHPLC column	Thermo Scientific (Waltham, MA, USA)	97502-102130
Orbitrap Fusion™ Lumos™ Tribrid™ Mass Spec	Thermo Scientific (Waltham, MA, USA)	IQLAAEGAAPFADBMBHQ
EASY-nLC™ 1200 System	Thermo Scientific (Waltham, MA, USA)	LC140
PepMap RSLC C18 (75 μm x 50 cm)	Thermo Scientific (Waltham, MA, USA)	ES803A
Acclaim PepMap 100 (75 μm x 2 cm)	Thermo Scientific (Waltham, MA, USA)	164946
Biopsy Punch	Integra-Miltex Instruments (Princeton, NJ, USA)	33-33
Leica TCS SP8	Leica Microsystems (Wetzlar, Germany)	TCS SP8
7500 Fast Real-Time PCR System	Applied Biosystems (Foster City, CA, USA)	-
Sep-Pak C18	Waters (Milford, MA, USA)	WAT020515
EVOS XL Core Imaging System	Thermo Scientific (Waltham, MA, USA)	AMEX1000
EVOM Voltohmmeter	Physiologic Instruments (San Diego, CA, USA)	
