# Supplementary material for: PGC-1α mediates a metabolic host defense response in human airway epithelium during rhinovirus infections
Source: Nat Commun. 2021 Jun 16;12:3669. doi: 10.1038/s41467-021-23925-z (PMC8209127; doi:10.1038/s41467-021-23925-z)
Supplement: Supplementary file 3 — Description of Additional Supplementary Files [file 41467_2021_23925_MOESM3_ESM.pdf]

Description of Additional Supplementary Files

File Name: Supplementary Data 1
Description: RAW data of shotgun proteomics performed on ALI cultures infected with HRV-C15 at 4, 12, 24 h post-infection. Data includes TMT labeling schemes, interquartile boxplot analyses, and proteins separated by time point.

File Name: Supplementary Data 2
Description: Metabolites identified by LC-MS from ALI cultures infected with HRV-C15 at 4, 12, 24 h post-infection. Benjamini-Hochburg correction was used to control for false discovery rate.
