# Supplementary material for: PGC-1α mediates a metabolic host defense response in human airway epithelium during rhinovirus infections
Source: Nat Commun. 2021 Jun 16;12:3669. doi: 10.1038/s41467-021-23925-z (PMC8209127; doi:10.1038/s41467-021-23925-z)
Supplement: Supplementary file 6 — Reporting Summary [file 41467_2021_23925_MOESM6_ESM.pdf]

nature research

Corresponding author(s): David Proud

Last updated by author(s): May 5, 2021

Reporting Summary

Nature Research wishes to improve the reproducibility of the work that we publish. This form provides structure for consistency and transparency in reporting. For further information on Nature Research policies, see our Editorial Policies and the Editorial Policy Checklist.

Statistics

For all statistical analyses, confirm that the following items are present in the figure legend, table legend, main text, or Methods section.

n/a	Confirmed	
☐	☒	The exact sample size (*n*) for each experimental group/condition, given as a discrete number and unit of measurement
☐	☒	A statement on whether measurements were taken from distinct samples or whether the same sample was measured repeatedly
☐	☒	The statistical test(s) used AND whether they are one- or two-sided<br>*Only common tests should be described solely by name; describe more complex techniques in the Methods section.*
☐	☒	A description of all covariates tested
☐	☒	A description of any assumptions or corrections, such as tests of normality and adjustment for multiple comparisons
☐	☒	A full description of the statistical parameters including central tendency (e.g. means) or other basic estimates (e.g. regression coefficient) AND variation (e.g. standard deviation) or associated estimates of uncertainty (e.g. confidence intervals)
☐	☒	For null hypothesis testing, the test statistic (e.g. *F*, *t*, *r*) with confidence intervals, effect sizes, degrees of freedom and *P* value noted<br>*Give P values as exact values whenever suitable.*
☒	☐	For Bayesian analysis, information on the choice of priors and Markov chain Monte Carlo settings
☒	☐	For hierarchical and complex designs, identification of the appropriate level for tests and full reporting of outcomes
☒	☐	Estimates of effect sizes (e.g. Cohen's *d*, Pearson's *r*), indicating how they were calculated

Our web collection on statistics for biologists contains articles on many of the points above.

Software and code

Policy information about availability of computer code

Data collection	No software was used.
Data analysis	GraphPad Prism (V.9.0) was used for most non-omics statistical analyses.<br>Proteomics analysis: Spectral data were matched to peptide sequences in the human UniProt protein database (https://www.uniprot.org) using the Andromeda algorithm as implemented in the MaxQuant57 software package (v.1.6.0.1). Data analysis was performed using Metascape (www.metascape.org).<br>Metabolomics analysis: Metabolite analyses were completed using El-Maven (v.0.12.0) , a mass spectrometry data analysis software package and metabolomic analyses and statistical figures were generated using MetaboAnalyst 5.0 (www.metaboanalyst.ca).<br>ImageJ (v. 1.52i)<br>Leica LASX (v.3.5.5.19976)<br>Xcalibur (v. 4.0.21.10)

For manuscripts utilizing custom algorithms or software that are central to the research but not yet described in published literature, software must be made available to editors and reviewers. We strongly encourage code deposition in a community repository (e.g. GitHub). See the Nature Research guidelines for submitting code & software for further information.

Data

Policy information about availability of data

All manuscripts must include a data availability statement. This statement should provide the following information, where applicable:

- Accession codes, unique identifiers, or web links for publicly available datasets
- A list of figures that have associated raw data
- A description of any restrictions on data availability

Source data associated with Figure and Supplemental Figure panels are provided with this paper as a Source Data file. Proteomics RAW data was deposited to ProteomeXchange via the Proteomics Identification Database (PRIDE) under accession number PXD02459. Metabolite LC-MS data was deposited to Metabolomics Workbench under accession number ST001774. Clinical HRV infection study data was obtained from the National Center for Biotechnology Information/Gene Expression Omnibus (GEO) accession number GSE11348.

Field-specific reporting

Please select the one below that is the best fit for your research. If you are not sure, read the appropriate sections before making your selection.

☒ Life sciences ☐ Behavioural & social sciences ☐ Ecological, evolutionary & environmental sciences

For a reference copy of the document with all sections, see nature.com/documents/nr-reporting-summary-flat.pdf

Life sciences study design

All studies must disclose on these points even when the disclosure is negative.

Sample size	For studies using cultured cells, all sample sizes were based on prior studies using cultured human airway epithelial cell where significant effects were demonstrated. (Example publications include: Proud D. et al J. Immunol. 172:4637, 2004; Spurrell J. et al. Am. J. Physiol. Lung Cell. Mol. Physiol. 289:L85, 2005; Hudy M et al. Am. J. Respir. Cell Mol. Biol. 50:571, 2014; Warner S, et al. Respir. Res. 20:150, 2019; Jamieson K et al. Front. Cell. Infect. Microbiol. 10:103, 2020). For clinical studies, sample size calculations were performed based on differences seen in prior studies, both from our lab and in the literature. (Example publications include: Sanders S et al J. Allergy Clin. Immunol. 107:235, 2001; Proud D. Am. J. Respir. Crit Care Med. 178:962, 2008; Kloepfer K et al. Ann. Allergy Asthma Immunol.106:252, 2011; Hansel T et al. EBioMedicine 19:128, 2017).
Data exclusions	No data exclusions were performed.
Replication	All data was obtained using multiple biological replicates of cells derived from individual human lung donors; each "n" represents one individual lung donor. Experiments were replicated using samples from 4-6 individual donors. Exact numbers for each individual type of experiment are provided in Figure Legends and in the Source Data file. All attempts at replicating results were successful.
Randomization	ALI cultures from human bronchial epithelial cells were differentiated in 12-well transwell plates for 5 weeks. During treatment allocation, transwells were transferred to new 12-well plates for stimulation in a randomized fashion. Treatments were randomly allocated within each plate. For the clinical genomics study, patients were randomized to receive either HRV-16 infection of sham inoculation using a randomization code generated via computer. For the study currently under progress (used for RNASeq analysis) two groups of patients were used. Healthy control subjects and cigarette smokers. These groups were chosen specifically as the purpose of the study was to compare immyne responses to HRV infection in smokers versus healthy subjects.
Blinding	Quantitative proteomics was performed blinded by a third party with respect to uninfected cells at each time point. Quantitative metabolomics was performed by a hired service (Calgary Metabolomics Research Facility) who were not aware of which samples were which. Cell counts for propidium iodide viability assays were performed blinded by a third party. All immunofluorescence data were captured by an individual blinded to treatment. For clinical studies, gene analysis were performed by labs that were blinded to sample identity.

Reporting for specific materials, systems and methods

We require information from authors about some types of materials, experimental systems and methods used in many studies. Here, indicate whether each material, system or method listed is relevant to your study. If you are not sure if a list item applies to your research, read the appropriate section before selecting a response.

Materials & experimental systems

n/a	Involved in the study	
☐	☒	Antibodies
☐	☒	Eukaryotic cell lines
☒	☐	Palaeontology and archaeology
☒	☐	Animals and other organisms
☐	☒	Human research participants
☐	☒	Clinical data
☒	☐	Dual use research of concern

Methods

n/a	Involved in the study	
☒	☐	ChIP-seq
☒	☐	Flow cytometry
☒	☐	MRI-based neuroimaging

Antibodies

Antibodies used

All antibodies used are commercially available.

Primary Antibodies:
ZO-1 (mouse monoclonal, clone ZO-1-1A12, Cat. 33-9100, Lot. T1275438, ThermoFisher Scientific) [1:200 IF]
ZO-1 (rabbit polyclonal, Cat. 40-2220, Lot. SA243690, ThermoFisher Scientific) [1:200 IF]
Occludin (mouse monoclonal, clone OC-3F10, Cat. 33-1500, Lot. RG230928, ThermoFisher Scientific) [1:200 IF]
dsRNA (mouse monoclonal IgG2a, Cat. J2-1504, Lot. J2-1801, Scicons) [1:200 IF]
beta-tubulin (mouse monoclonal, clone D66, Cat. T0198, Lot. 035M4782V, Sigma Aldrich) [1:1000 IF, 1:500 IHC]
beta-tubulin (rabbit monoclonal, 9F3, Cat. 2128, Cell Signaling Technology) [1:1000 IF]
PGC-1alpha (rabbit monoclonal, clone 3G6, Cat.2178, Lot. 4, Cell Signaling Technology) [1:1000 WB]
GAPDH (mouse monoclonal, Cat. MCA 4739, Lot. AbD Serotec) [1:10,000 WB]

Detection Antibodies:
AlexaFluor488 (conjugated goat anti-mouse IgG (H+L), Cat. A11029, Lot. 1789729, ThermoFisher Scientific) [1:200 IF]
AlexaFluor488 (conjugated goat anti-rabbit IgG, Cat. A11034, ThermoFisher Scientific) [1:200 IF]
AlexaFluor647 (conjugated goat anti-mouse IgG (H+L), Cat. A32728, Lot. SA243835, ThermoFisher Scientific) [1:200 IF]
HRP-linked anti-rabbit IgG (Cat. 7404, Lot. 27, Cell Signaling Technology) [1:10,000 WB]
HRP-linked goat anti-mouse IgG (Cat. 115-035-003, Lot. 117228, Jackson ImmunoResearch Laboratories) [1:10,000 WB]
Biotinylated horse anti-mouse (Vectastain ABC Kit, Cat. PK-4002, Lot. ZG0916, Vector Laboratories) [1:200 IHC]

Validation

All antibodies were used at concentrations and conditions recommended by the manufacturer. In addition to manufacturer validations, we performed matched isotype controls (IgG) for each experiment.

ZO-1 (33-9100): validated for human IF by siRNA mediated knockdown (Caco-2 cells)
ZO-1 (61-7300): validated for human IF (Caco-2 cells), not validated but recommended for IF Occludin (33-1500): validated for human IF by IL-1B treatment to reduce expression (Caco-2 cells)
dsRNA (J2-1504): validated for IF (Schönborn J, et al. Nucleic Acids Res. 1991)
beta-tubulin (T0198): validated for IF (SH-SY-5Y cells), not validated but recommended for IF
PGC-1a (2178): validated for WB by construct transfection and PGC-1a overexpression (293T cells), validated in-house by siRNA mediated knockdown of PGC-1a in primary human bronchial epithelial cells.
GAPDH (MCA 4739): validated for WB (HeLa cells).

Eukaryotic cell lines

Policy information about cell lines

Cell line source(s)	All cell lines and their sources are listed in Supplementary Table 3.
Authentication	Caco-2 cells were not formally authenticated in our laboratory.
Mycoplasma contamination	All cell lines tested negative for mycoplasma contamination.
Commonly misidentified lines (See ICLAC register)	No such lines were used.

Human research participants

Policy information about studies involving human research participants

Population characteristics	For the experimental rhinovirus infection study published in 2008, healthy normal subjects aged 18-60 were used as described in the online supplement to the paper. The normal subjects analyzed for gene expression in the ongoing study were healthy normal subjects aged 18-65 years. Human lung tissues that were not used for transplant were obtained from otherwise healthy subjects (aged 13 to 65) who died of head trauma of cerebrovascular disease.
Recruitment	Human lung tissues were obtained by a non-profit tissue retrieval service. Volunteers for experimental rhinovirus infection studies were recruited using advertisements approved by the appropriate Ethics Board detailed above. As noted above, Human lung tissues that were not used for transplant were obtained from otherwise healthy subjects (aged 13 to 65) who died of head trauma of cerebrovascular disease. There was no bias in subject selection except for information on the healthy quality of the lungs obtained. This was assessed based on patient history (no identifiers of the subject were provided) quality of lung X-rays or chest CT and infection status or high risk behaviour (e;g; current iv drug abuser etc).
Ethics oversight	The study published in 2008 was reviewed and approved by the Human Investigations Committee of the University of Virginia. The ongoing study of experimental human rhinovirus infection was approved both by the Conjoint Health research Ethics Board of the University of Calgary and by Health Canada. The Conjoint Health research Ethics Board of the University of Calgary approved obtaining human lung tissue for isolation of epithelial cells.

Note that full information on the approval of the study protocol must also be provided in the manuscript.

Clinical data

Policy information about clinical studies

All manuscripts should comply with the ICMJEguidelines for publication of clinical research and a completedCONSORT checklist must be included with all submissions.

Clinical trial registration	Not Applicable. Both of the clinical studies conducted were investigator initiated studies to examine transcriptomic and immune responses to HRV infections. They were not clinical trials as no interventions were used, so clinical trial registration was not required.
Study protocol	The trial protocol for the 2008 study is described in the Supplement to that paper. The Protocol for the ongoing study is available from the authors.
Data collection	All samples were collected in clinical research suite with full emergency equipment and samples were processed in our research laboratories.
Outcomes	Clinical outcomes were not assessed for the purposes of the transcriptomic analyses performed. We only examined gene expression of a single gene before and after experimental HRV infection.
